# Patients’ Participation in Health Research: A Classification of Cooperation Schemes

**DOI:** 10.2196/jopm.8933

**Published:** 2017-10-12

**Authors:** Olivier Las Vergnas

**Affiliations:** ^1^ Centre InterUniversitaire de Recherche en Education de Lille Education and Adult Training Department University of Lille Villeneuve d'Ascq France; ^2^ Centre de Recherche en Education et Formation University of Paris–Nanterre Nanterre France

**Keywords:** patient engagement, typology, bibliometrics, popular epidemiology, patient organization

## Abstract

**Background:**

The number of academic papers referring to patient engagement or to related terms has been rising sharply for at least 20 years; several review articles have recently been published enumerating a wide variety of situations of patient involvement in research and partnership with health professionals.

**Objective:**

As no standardized keywords and no shared classifications exist to facilitate comparative studies of situations where patients and their organizations are recognized as coresearchers, this paper purports to create a typology to analyze those situations.

**Methods:**

Based on 8 already existing meta-reviews or related studies, this work is achieved using a template based on Claude Bernard’s conceptualization about experimental medicine.

**Results:**

This typology allows differentiating between modes of involvement and levels of patients reflexivity mobilized in evidence-based medicine (EBM) trials. Screening through a first set of various meta-reviews using this typology shows that a high level of reflexivity is seldom observed and seen only when a patient organization (PO) is involved in the process. This suggests that such an organization can play several roles essential to high reflexivity trials; the PO is capable not only of grouping singular approaches but also of synchronizing and correlating them. However, as nowadays health researchers and POs give more attention to syndromes or troubles for which EBM clinical trials are not relevant due to lack of biomedical indicators (eg, fibromyalgia, chronic fatigue syndrome, or psychiatric disorders), a supplementary mediation category is added to take into account action-research, community-based participatory research, and grounded theories.

**Conclusions:**

With this new category, this typology should be able to classify most of the cooperation schemes and thus be a useful tool for the next systematic reviews.

## Introduction

**Figure 1:**
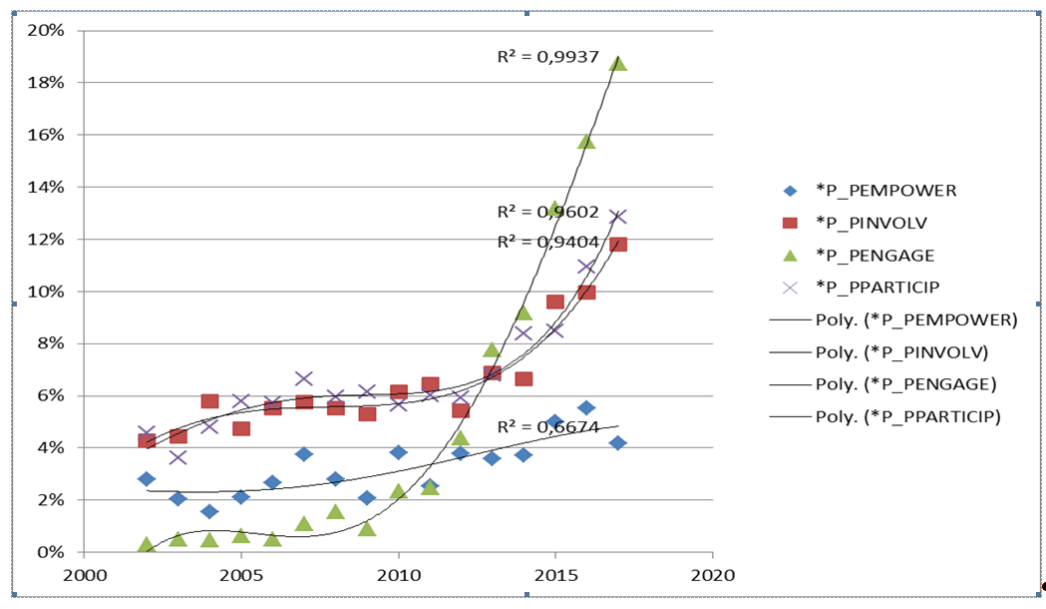
Ratios of publications mentioning patient commitment, patient involvement and patient participation compared to those mentioning pneumothorax and psoriasis in their titles and abstracts according to PubMed data. The slower growth of the patient empowerment ratio was also indicated as a reference. All curves are approximated by 4th degree polynomials (Microsoft Excel).

The number of publications mentioning patient engagement and related terms such as patient involvement or patient participation reported through PubMed has strongly increased for at least 2 decades, as shown on Figure 1.

Screening those publications shows that the uses of the terms patient engagement, patient involvement, and patient participation have developed concomitantly over the last few years without any clearly defined differences in acceptance. Most works use one of these terms without specifying why they have chosen it, either because authors consider them synonymous or because they do not feel the need to justify their choice. More generally [1], bibliometric approaches to those issues reveal weak efficiency and relevance due to a lack of shared keywords linked to a good typology that could enable involvement in protocols to be compared. This is confirmed by the work of Domecq et al [2], which shows that among the 5551 recent papers mentioning patient engagement, only 142 give useful data about the way those patients have been involved in protocols.

It results from this situation that, although the recognition of patient experiential knowledge (PEK) has reached the level of a social fact attested by diplomas, jobs, laws, and academic concepts [3], no standardized keywords and no shared typology can actually be used to facilitate comparative studies of situations where patients and their organizations are recognized as coresearchers. As Domecq et al [2] have recommended that “bibliographic databases use indexing terms that identify active patient engagement in research to facilitate future research in this field,” it is obvious that progress requires methods and typologies to describe which role PEK plays in health research and how to reduce the risks of tokenism.

In this context, this paper aims at creating a classification embracing patient opinions and approaches as well as those in which patient contributions based on their PEK are accepted in their own right (ie, where patients are fully accepted as coresearchers).

From a methodological point of view, without shared keywords, ascendant clustering converging to a semantic-based taxonomy is not possible. Therefore, our aim cannot be to create a taxonomy based on a bibliometric or lexical study but rather to formalize a complete classification (a set of rational categories)—that is to say, a categorization which allows us to specify major types corresponding to the main ways in which patients and academic researchers associate today.

## Methods

### A Typology Able to Indicate Different Levels of Patient Reflexivity

Thanks to previous studies [3], we began this work intending to distinguish at least 2 categories characterized by different levels of patient reflexivity: on the one hand, cooperation in which patients were only associated with data collection, and on the other hand, cooperation in which they were associated also with design or conclusion. Thus, we imagined a working method in 2 steps, aimed at obtaining a typology validated by a completeness test.

- Step 1: Look for a template or grid able to describe the phases in which the cooperation would be mobilized or not (we used a very simplistic description of Claude Bernard's experimental medicine [4]).
- Step 2: Using this model, screen a corpus of papers embracing the broadest types of patient cooperation with 2 aims: validate the best set of categories capable of accounting for the diversity of cooperation encountered and be sure that any kind of cooperation could fit in one of those categories.

Due to the keywords issue, instead of creating a new minute, even pernickety, review of primary literature, we decided for step 2 to use already published meta-reviews such as those quoted above. Our first idea was to select them through a systematic search on PubMed. However, similar problems of terminology relevance arose. Queries on PubMed titles or abstracts for review and patient engagement (152 reviews found), patient involvement (246) or patient participation (275) provide too numerous results; 642 different reviews are found with 1 of those 3 terms. Therefore, we opted for a pragmatic, targeted selection and chose a small set of 8 complementary meta-reviews that were already often quoted:

- Four general syntheses and descriptions concerning situations of coresearch found in medical literature [1,2], cases of PEK recognition [3], and training partnership actions [5]
- Three more specific meta-reviews: autoethnography [6] and mental health user involvement [7,8]
- An eighth review paper dedicated to inventory and description of the European Patients’ Organizations in Knowledge Society (EPOKS) [9]

We added this last review paper because we wished to take both collective and individual cooperation into account.

### A Template Inspired From Claude Bernard’s Model

As explained previously, our first goal was to be able to distinguish different levels of reflexivity recognized by patients; therefore, an easy-to-use description of current research protocols was needed. To build a first version of the typology itself, we elected to use a system of description inspired from a simplification of Claude Bernard’s formalization of experimental medicine [4]; this idea was first presented in Jouet et al [3]. This model, called OHERIC, divides investigations into a pragmatic grid: initial Observation, Hypothesis, Experiment proper, Result, Interpretation, and Conclusion (see Table 1).

This OHERIC grid is introduced only to be used in a quite pragmatic way as an ideal type description or a computational intermediate (ie, a background against which to set the practices we aim at describing). In view of the critic of linearity, we give no specific chronological significance to OHERIC phases, which can then be considered as aspects of the same process that can overlap or even interpenetrate.

The OHERIC pattern can be used to build grids in which arrows specify who exerts the main reflexivity at each stage of the research. Up arrows specify a nonacademic origin (bottom-up process), while down arrows designate an academic origin. This appears close to the stages of research process as described in the *Handbook of Service User Involvement in Mental Health Research* [8], as is shown in the third line of Table 1. Referring to autoethnography led by a patient [6], the fourth line shows that OHERIC can also be compared to Dewey’s self-inquiries [10].

**Figure 2:**
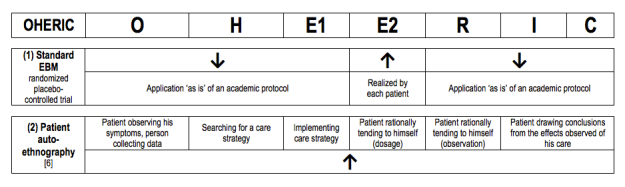
Comparison between 2 protocols for chronic diseases.

Figure 2 shows how this encoding allows comparing 2 such protocols: an evidence-based medicine (EBM) randomized placebo-controlled trial and an autoethnography.

**Table 1:** Phases of an investigation described with the stages of Bernard, Wallcraft, and Dewey.

OHERIC phases	O^a^	H^b^	E^c^		R^d^		I^e^		C^f^	
Bernard’s research phase [4]	Initial data or observation leading to research	Putting forward a research hypothesis	Building an experimental device, inventing a test	Collecting observations, realizing a test		Processing results and data		Interpreting results		Scientific conclusion
Walcraft et al “Involvement in Mental Health Research”	Identifying a research topic	Designing research	Outcomes measures	Data collection		Data analysis		Interpretation		Write up and dissemination
Dewey’s phenomenology “How we think”	A felt difficulty; its location and definition	Suggestion of a possible solution	Development by reasoning of the bearings of the suggestion	Further observation and experiment		Leading to its acceptance or rejection (1)		Leading to its acceptance or rejection (2)		That is, the conclusion of belief or disbelief

^a^O: initial observation.

^b^H: hypothesis.

^c^E: experiment proper.

^d^R: result.

^e^I: interpretation.

^f^C: conclusion.

## Results

### First Typology Distinguishing Low and High Reflexivity Engagement

Such a template can easily be applied to the clinical trials in EBM because they are based on protocols that are close to the OHERIC phase sequence. Therefore, it is quite easy to create a first classification defining different levels of mobilization for the patients’ reflexivity. Concerning patients associated with this kind of trial, OHERIC grids allow us to distinguish 2 main types of roles: (1) patients and their relatives were mainly considered as mere data collectors and (2) more diverse situations that all had a fact in common—patient reflexivity (ie, PEK) was at the heart of the research process.

More precisely, category 1 corresponds to an involvement of patients and relatives as data collectors in the E phase of OHERIC. Collected data may consist of personal opinions and biomedical parameters (eg, blood pressure, glycemic measurements) or self-evaluation according to one’s own perception (eg, pain, anxiety, discomfort) or opinion [11,12].

In some cases belonging to this first category, patients are also associated with part of the processing of the data collected (OHERIC R). As this has the effect of reinforcing their reflexive activity, we decided to characterize these situations through a new category we named 1+. At this step, we can mention that it could be relevant to distinguish the collection of opinions from the collection of biomedical parameters (either quantitative or qualitative) through 2 more subcategories 1_OP_ (opinions) and 1_PA_ (parameters). We will see later that this first subcategory, 1_OP_ (opinions), has to be merged into a larger one (ie, M category).

Category 2 collects situations where patients and relatives contribute with their reflexive capacities to other phases besides data collection or initial processing. We first distinguished 2 subcategories in it: 2 for participatory EBM and 2+ for a full popular epidemiology process.

The subcategory 2 or 2_ParEBM_ was created to characterize situations corresponding to what we can call participatory EBM where academics decide to involve—in parallel to the main EBM process—lay people’s reflexivity for all research phases following the initial set-up (ie, all OHERIC but O or perhaps E).

**Figure 3:**
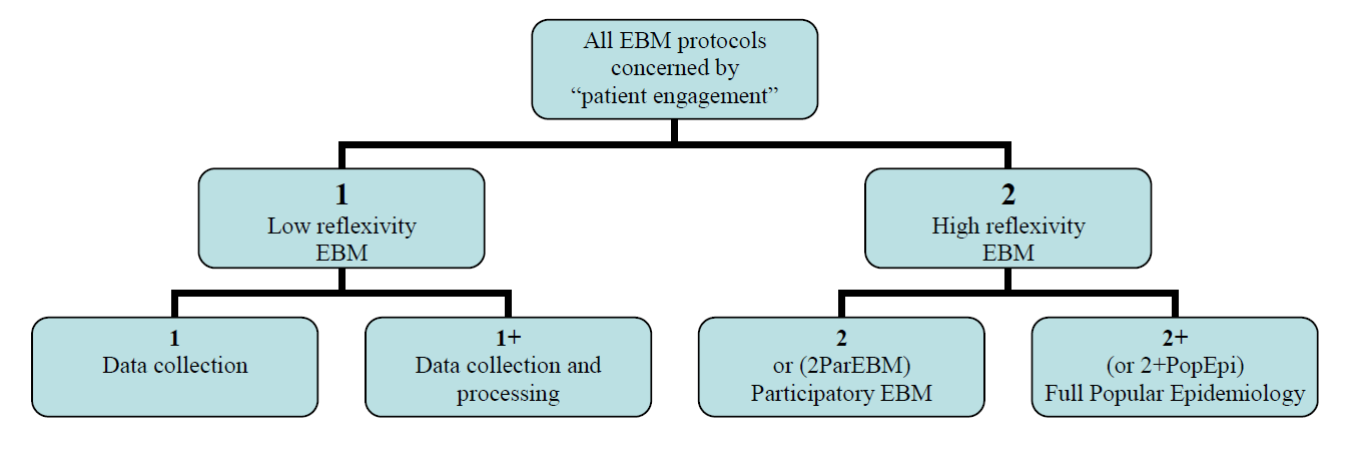
Categories and subcategories of types of involvement forpatients and relatives as coresearchers in medical research projects.

The second subcategory, 2+ or 2+_PopEpi_, is inspired by Brown’s research in medical sociology [13]; Brown has introduced the term popular epidemiology in reference to parents and teachers confronting a cluster of childhood leukemia (the Love Canal School case, named after a polluted area near Niagara Falls) who could keep control as well over the subsequent phases. Such autonomous grasp (OHERIC) of a medical problem by a concerned community obviously raises issues concerning differences in social representation of expertise [14]. Figure 3 summarizes those categories and subcategories.

### Findings Concerning Patient Organizations

#### The Significant Role of Patient Organizations in High Reflexivity Cooperation

Screening the publications quoted in meta-reviews shows that individual patients are seldom recognized as contributing fully to the reflexive production of new knowledge. As a matter of fact, in category 2, real research responsibilities are entrusted to collective community actors rather than to individual patients, who find themselves restricted to type 1 functions (ie, data collection). Such intermediary collective actors may be whole communities, mutual assistance groups, or patient organizations (POs).

By providing a framework for collective action and investigation, these POs not only help patients develop their individual reflexivity and ability to compare their situations but also lead patients to synchronize them. Collective, synchronized patients’ reflexivity and investigations become then more readily describable in the language of a collective protocol taking on the form of a succession of stages akin to the OHERIC formalism.

If we adopt the spiral-shaped representation of the individual pragmatic forms of thinking (in the sense of the curls proposed—in addition to Dewey’s work—by Ashby [15]), we can consider that these POs carry out a work of standardization and of unfolding those individual curls, in a process similar to an uncurling or a straightening.

Furthermore, once started, such a PO producing an OHERIC-type protocol aggregates the incoming patients by putting them literally in sync with those already included. And it is precisely because the work accomplished by the PO is then collectively turned into hypothetical-deductive parlance thanks to this unfurling that it is made acceptable in type 2 projects without endangering the criteria of the academic patterns according to which new medical knowledge is produced. POs then fulfill a role of socializing and reformulating each individual patient’s metacognition and reflexivity, allowing them to be taken into account by academic research teams as intellectual inputs. Furthermore, these POs may also be places where collective inquiries can be decided, either invented by the patients themselves or suggested by relatives, caregivers, clinicians, or researchers.

**Figure 4:**
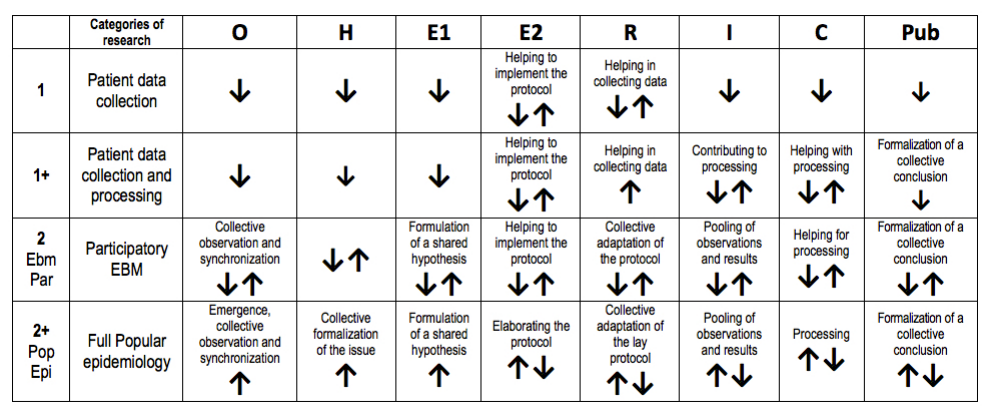
Potential effect of a patient organization according to research categories.

The collectivization of reflexivity operated by a PO can act during different OHERIC phases: Observation and Hypotheses, through the collective problematization or formalization of the issue (this includes issuing a hypothesis that can be tested through collectively taking/acquiring a critical distance from situations experienced individually); Experience and Results, as a self-training framework in which patients learn how to observe and tend to themselves (and sometimes as a furnisher of note-taking tools, multiple choice questionnaires, or quantified self-tools); and Interpretation and Conclusion, by organizing the formalization of conclusions. Figure 4 abstracts the different roles played by POs and locates them along OHERIC phases.

#### A Three-Layer Point of View: Patients’ Dewey Pragmatics, Bernard’s OHERIC, and Evidence-Based Medicine Trials

From an epistemological point of view, it can be said that the multiple interactions between various patients through a PO bring about a reshaping of their singular pragmatic phenomenology into a kind of investigation relevant for EBM researchers (eg, in a collective process that fits with Bernard’s experimental medicine). Therefore, we may represent interactions between the patients and the academic researchers using a 3-layer model: pragmatic phenomenology (Dewey’s level) is linked to experimental method (Bernard’s level) through PO interaction, and these 2 levels are themselves linked to the third one (EBM clinical trials). Textbox 1 shows the superposition of these 3 layers. It shows the role of the POs between patient pragmatic phenomenology and EBM in type 2 or 2+.

**Three layers model for 1 and 1+ or 2 and 2+ types.**

Level of global scientific research:

Clinical trials developed in evidence-based medicine epistemology [16]

Level of rational work, local or in group:

OHERIC-describable protocols [4]; work in a patient organization may allow relating experiential knowledge acquired through pragmatic phenomenology to evidence-based medicine protocols

Level of individual experiential knowledge:

Dewey’s pragmatic phenomenology of each patient [10]

#### High Reflexivity Without Patient Organizations?

In the EBM clinical trials point of view [16], this led us to the conclusion that patient reflexivity and lay production of knowledge are taken in account by EBM clinical trials only if their results are adapted or translated into a format allowing their description through an EBM protocol. The uniquely possible agents of this translation seem to be those POs that indeed appear to play an intermediary role, socially as well as epistemologically; without any PO to translate between individual pragmatism and OHERIC protocol, academic researchers stick to category 1, asking patients to bring data in phases ER, doing the work in phases OH and IC themselves. They tend not to engage in leading patients through category 2, a work they may consider unnecessary or outside their capacities. They may, however, set up devices to listen to patients’ voices [1,2,11,12] or train patients as cotrainers and peer experts [3].

However, when researchers following EBM protocols realize they need to take into account not only biomedical data but patients’ reflection as well, they themselves may try to foster the creation of POs, either in cooperation with the patients or with economic actors as manufacturers of health products or health insurers. Meta-reviews show that with the great expansion of interest in mobile phones and other connected objects, numerous cooperation programs have now been established between researchers, mHealth companies, and POs, either preexisting or specially created for this purpose.

### Addition of a Mediation Category

#### High Reflexivity Outside or Beyond Evidence-Based Medicine Clinical Trials

Of course, cooperation between patients and academic researchers is not limited to the pattern of clinical trials in EBM. The existence of other cooperation schemes is easy to verify through an analysis of more specific corpora (ie, mental health [7,8] or autoethnography–oriented [6] reviews). Screening recent reviews about patient engagement shows that eHealth or mHealth new cooperative programs go far beyond the scope of EBM clinical trials. As we open our field of observation to human sciences disciplines such as sociology, anthropology, or ergonomics, it is easy to observe that participatory situations of knowledge construction are frequent and very diverse.

In fact, the POs have different postures regarding cooperation with health researchers, and EPOKS’s evidence-based activism [9] or Epstein’s impure science [14] are describing other schemes besides a simple allegiance to EBM clinical trials. Nowadays, some of the POs choose to adopt the posture that we described in Figure 3. This may cover both the 2 and 2+ categories, depending on whether the POs were the promoter of the protocol: it is the case in 2+ (OHERIC where PO promotes the research and then associates with medical research teams or even mHealth apps or services) and not in 2. But in fact, many other POs do not focus on promoting the collective comparison and reflection on individual situations to bring data to EBM. On the contrary, several POs (for instance, those concerned with psychiatric disorders or syndromes not directly linked with biomedical indicators) criticize what they see as too narrow a conception of knowledge coproduction in EBM (ie, an exclusively positivist anchorage of EBM built on the idea that evidence must only result from an experimental procedure related to hypothetical-deductive assumptions).

In an intermediate posture between what we can call allegiance to the EBM and radical opposition to it, many POs are focused on individual strategies and folk theories and on developing intersubjectivity through the transformation of individual experiences into narratives or accounts that can be shared.

#### M Category as a Way to Go Beyond the Evidence-Based Medicine Research Limitation

As a matter of fact, when data consist only of isolated patients’ dispersed and unsynchronized lived accounts, EBM (particularly clinical trials) has the effect of drastically limiting the potential contributions of patient reflexivity to the construction of new health knowledge (see, for instance, Faulkner and Thomas [16] for mental health). If phenomena can be observed only through the perceptions of the patients and their relatives (as is the case for anxiety or pain in cases such as fibromyalgia, for instance) and treatment efficiency cannot be studied without listening to them, researchers need to open more opportunities for the bottom-up transmission of patients’ lived-through experiences, however different and even heterogeneous they may appear. How can researchers bring such reflexive materials to convergence not only in case-by-case individual experiential knowledge but also in a corpus that can be used to produce innovative health knowledge?

We thought it necessary to distinguish between these different cases while still retaining the already mentioned 2, in which we had specifically included EBM research projects organized beforehand to entrust patients with specific functions that can be described through a OHERIC framework (eg, a patients’ group organizing and analyzing specific biographic workshops to collect qualitative data in order to compare various evaluation processes of the effectiveness of a long-term treatment on pain perception).

Hence we decided to define, besides the main categories 1 and 2, a third category called M (as for mediator in the sense of facilitators) to collect cases where patients were used as intermediaries, facilitators, or even multipurpose interpreters [17]. With such a wide definition of our M category, any kind of research dedicated to collect only patients’ opinions (but not feelings) belongs to M type, and category 1_OP_ turns out to be useless, as it appears to be one of the M subcategories.

For that purpose, M ethnological protocols offer an alternative to the Dewey-OHERIC-EBM translation. It relates to the patients in both a more heuristic and comprehensive manner, as is shown in Table 2.

Practically, patients are no longer seen only as witnesses bringing data to hypothetical-deductive epistemologies. Constructions of folk and academic types of knowledge can then be put into relationship under the condition that other tools are brought to the core of the protocols: tools of description of patients’ relationships to their illness as well as tools of production of innovating knowledge, using not only hypothetical-deductive models but also more comprehensive methods, such as grounded theory [18], for instance.

#### Recognizing Coresearchers Outside of Evidence-Based Medicine

The recourse to ethnological protocols is not the only approach found by researchers to short-circuit the Dewey-OHERIC-EBM translation and allow the setting up of other bottom-up chains of production of knowledge. Other disciplines are used in M category to blend with the benefits available from patients’ and relatives’ pragmatic phenomenology in order to achieve more efficiency and hence more confidence and observance from its beneficiaries. This is, for instance, the case with educational science for research on PEK [3], ergonomics for research on patients’ voices [2] or patient preferences [19], and, of course, information-communication for numerous studies of eHealth.

**Figure 5:**
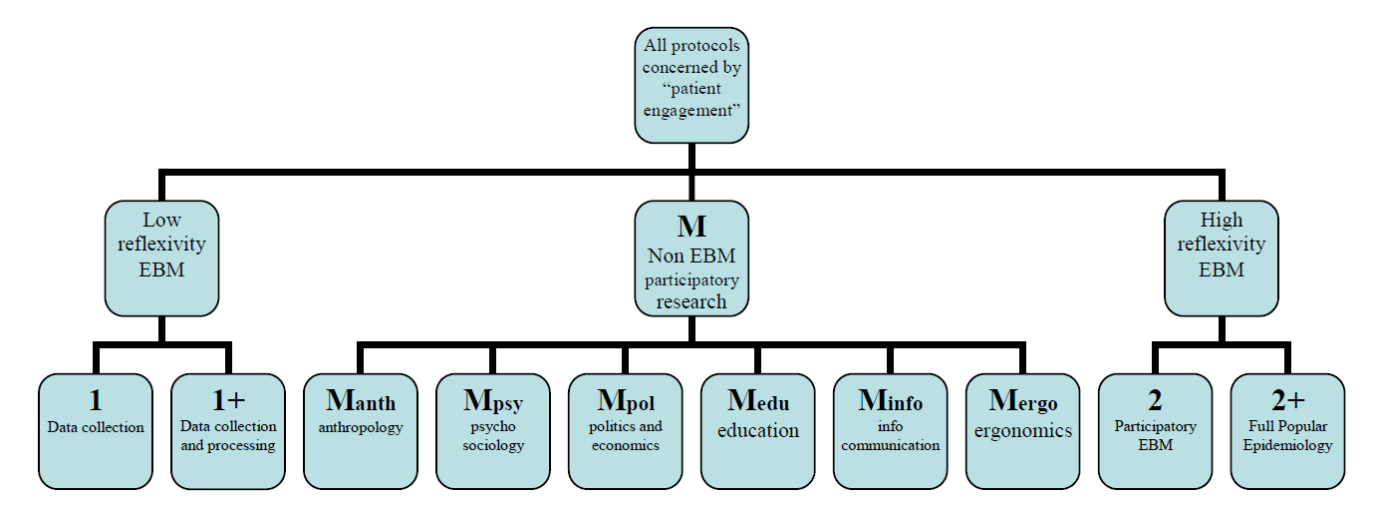
Completed typology tree.

Table 3 is a complement to Figure 3, focusing on M category. It gives a list of those nonmedical disciplines (this list is only indicative as several other epistemologies can also contribute to new knowledge production in health), and Figure 5 shows a refined tree of categories.

**Table 2:** Three layers model used to compare 2 regimes of connection between pragmatic phenomenology and global knowledge production.

Coupling model	EBM^a^ clinical trials regime through patient organizations and Claude Bernard	Full participatory action research non-EBM regimes
Types	1, 1+, 2, 2+	M
Level of global scientific research	Epistemology of the EBM [16]	Epistemologies of comprehensive research (such as action research [17] or grounded theories [18])
Level of rational work, local or in group	OHERIC^b^-describable protocols [4]; work in a patient organization may allow relating experiential knowledge acquired through pragmatic phenomenology to EBM protocols	Comprehensive organization through participatory M category action research
Level of individual experiential knowledge	Dewey’s pragmatic phenomenology of each patient	Pragmatic phenomenology of each patient

^a^EBM: evidence-based medicine.

^b^OHERIC: initial Observation, Hypothesis, Experiment proper, Result, Interpretation, and Conclusion.

**Table 3:** Examples of subcategories for M type based on disciplines contributing to knowledge production in complement to evidence-based medicine.

M sub- categories	M anthropology	M psycho-sociology	M politics and economics	M education	M info-communication	M ergonomics
Concepts, facts studied	Culture, ethics, knowledge, and beliefs	Social representations	Health governance, users’ representation, health democracy, activism	Knowledge, abilities, self-education, learning	Connected tools, networks, quantified self, patient 2.0, eHealth	Adaptation of artifacts, prosthesis, customer made

## Discussion

Building such a typology also addresses the question of the social representations of research. Our hypothesis is that these representations are dependent on the need to associate lay people as producers of data, producers of PEK, and even as coresearchers: the greater the need to involve communities to obtain scientific results by using their (individual and collective) reflexivity, the greater the resulting shift by research bodies toward epistemologies more open to taking into account lay people’s pragmatic phenomenology.

Health research as a social construct negotiated among stakeholders: with the rise of impure science [14], evidence-based activism [9], and recognition of PEK [3], researchers can no longer remain confined in their ivory towers. Academic imperatives are not only exposed to the negative influence of economic issues but also to positive activist irruption by the concerned communities and even to their necessary involvement in the process: the more the objects of study also become subjects of studies and express their demands that the reflexivity of lay people be listened to (eg, studies on perceptions, feelings, representations), the more the epistemologies must adapt and accept their own articulation with the concerned people’s pragmatic phenomenology.

The main question is neither to determine whether academic knowledge obtained via clinical trials in EBM is worth more than patient knowledge experienced from synchronization of singular phenomenologies nor to choose the best model of knowledge production between Bernard, Dewey, or Lewin; on the contrary, it is now to find ways to make all those contributions converge. The more researchers must steer between the taken-for-granted representations of the world of lived experience and the ideal types of proof-finding, the more they will have to balance multiple ways to define how health research can be done.

## Abbreviations

EBM: evidence-based medicine
EPOKS: European Patients’ Organization in Knowledge Society
OHERIC: initial Observation, Hypothesis, Experiment proper, Result, Interpretation, Conclusion
PEK: patient experiential knowledge
PO: patient organization

## Appendix

Multimedia Appendix 1 Lexical analysis illustration of PubMed papers about "patient engagement" obtained by the author (Olivier Las Vergnas) using Iramuteq and R open source softwares.
