# Supplementary figures and images for: Patients’ Participation in Health Research: A Classification of Cooperation Schemes
Source: J Particip Med. 2017 Oct 12;9(1):e16. doi: 10.2196/jopm.8933 (PMC8080474; doi:10.2196/jopm.8933)

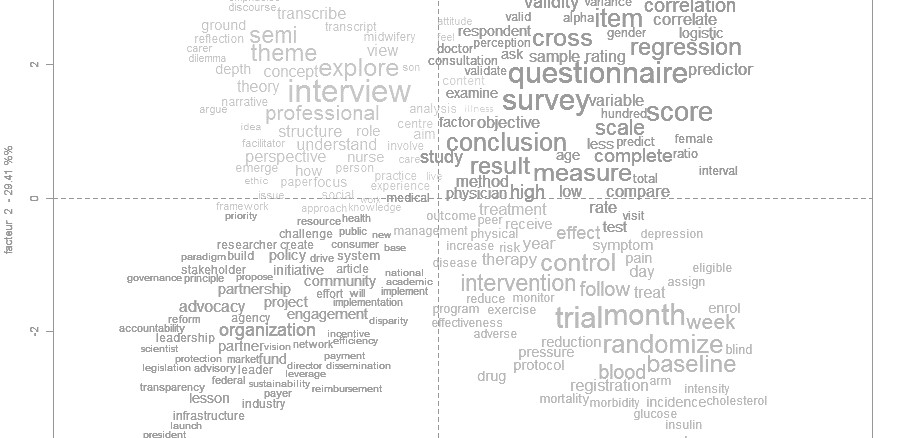

Supplement: Multimedia Appendix 1 [file jopm_v9i1e16_app1.jpg]
